# Frailty and prognosis of patients with kidney transplantation: a meta-analysis

**DOI:** 10.1186/s12882-023-03358-0

**Published:** 2023-10-13

**Authors:** Jianming Zheng, Yu Cao, Zhen Wang, Yeqi Nian, Liping Guo, Wenli Song

**Affiliations:** Department of Kidney and Pancreas Transplantation, Tianjin First Central Hospital, Nankai District, Tianjin, 300192 China

**Keywords:** Kidney transplantation, Frailty, Mortality, Meta-analysis

## Abstract

**Background:**

The prevalence of frailty among candidates and recipients of kidney transplantation (KT) is well-established, yet the impact of frailty on clinical outcomes following KT remains uncertain. To address this knowledge gap, we conducted a systematic meta-analysis to comprehensively assess the aforementioned relationship.

**Methods:**

The present study conducted a comprehensive search of PubMed, Embase, and Cochrane Library databases to identify relevant observational studies that compared mortality risk and other clinical outcomes of KT recipients with and without frailty. Two authors independently conducted data collection, literature searching, and statistical analysis. The results were synthesized using a heterogeneity-incorporating random-effects model.

**Results:**

In this meta-analysis, 6279 patients from 13 cohort studies were included, and 1435 patients (22.9%) were with frailty before KT. There were higher mortality rates among frail patients at admission, compared to those without frailty (risk ratio [RR]: 1.97, 95% confidence interval [CI]: 1.57 to 2.47, p < 0.001; I^2^ = 19%). Subgroup analysis suggested the association between frailty and high mortality risk after KT was consistent in studies of frailty assessed via Physical Frailty Phenotype or other methods, and in studies of follow-up duration < or ≥ 5 years. In addition, frailty was associated with higher incidence of delayed graft function (RR: 1.78, 95% CI: 1.21 to 2.61, p = 0.003; I^2^ = 0%), postoperative complications (RR: 1.88, 95% CI: 1.15 to 3.08, p = 0.01; I^2^ = 0%), and longer hospitalization (RR: 1.55, 95% CI: 1.22 to 1.97, p < 0.001; I^2^ = 0%).

**Conclusion:**

Following KT, frail patients are at higher risks for all-cause mortality, delayed graft function, postoperative complications, and longer hospital stays.

## Introduction

Kidney transplantation (KT) is a highly favored treatment modality for patients diagnosed with end-stage renal disease (ESRD) due to its ability to reduce mortality rates and enhance the quality of life, in contrast to renal replacement therapies like dialysis [1, 2]. The aging population and advancements in surgical techniques for KT have contributed to a significant increase in the number of older patients receiving KT in the United States, with data indicating a tripling of annual KT patients between 1998 and 2016 [3]. Although KT is generally effective for patients with ESRD, the response and prognosis of the recipients of transplant varied, which highlights the importance of identification of prognostic predictors in these patients [4–6]. As a geriatric syndrome, frailty is characterized by decreased reserves of various systems and a diminished ability to cope with stress [7, 8]. In older patients with various clinical conditions, frailty has generally been linked to poor prognosis and high mortality [9]. A recent systematic review and meta-analysis showed that for kidney transplant recipients, one in six is of frailty before transplantation [10]. However, the influence of frailty on mortality of patients after KT has not been summarized in a meta-analysis [11]. Our goal in this study was to evaluate the influence of frailty on the incidence of mortality from all causes and other clinical outcomes in patients after KT.

## Materials and methods

The present meta-analysis adhered to the guidelines set forth by the Preferred Reporting Items for Systematic Reviews and Meta-Analyses (PRISMA) statement [12, 13] and the Cochrane Handbook [14]. Furthermore, the meta-analysis protocol was registered with the International Platform of Registered Systematic Review and Meta-analysis Protocols (https://inplasy.com) under the registration number INPLASY202340008.

### Literature retrieving

A systematic search was performed in PubMed, Embase, and Cochrane Library to identify pertinent cohort studies from the inception of the databases until January 5, 2023. The search strategy employed the following combined terms: (1) “frailty” OR “frail”; (2) “renal” OR “kidney”; and (3) “transplantation” OR “transplant”. Our review exclusively considered clinical studies published in peer-reviewed journals in full-length in English. Additionally, we conducted a manual examination of the references of relevant original and review articles to identify potential studies of interest.

### Study selection

The present study employed PICOS-based inclusion criteria. Patients scheduled for KT, regardless of etiology and surgical protocol, were included as P (patients). Exposure was defined as frailty at admission, consistent with the modalities utilized in the original studies, and denoted as I. Patients without frailty at admission were designated as C (control). The primary outcome (O) of interest was all-cause mortality during follow-up, while secondary outcomes included the incidence of delayed graft function (DGF), overall postoperative complications, and proportion of patients with extended hospitalization compared between patients with and without frailty at baseline. The type of the studies (S) included in the meta-analysis were prospective or retrospective cohort studies. Within the context of this meta-analysis, DGF was operationally defined as the requirement for dialysis within the initial week following KT. The criteria utilized to define postoperative complications were based on the original studies. A prolonged hospital stay was defined as duration of hospitalization exceeding two weeks.

Exclusion criteria for the meta-analysis included reviews, meta-analyses, studies that did not involve patients receiving KT, studies that did not assess frailty, and studies that did not provide relevant outcome measures. In cases where patient populations overlapped, the study with the largest sample size was selected for inclusion in the meta-analysis.

### Data collection and quality assessment

The literature searches, data collection, and quality assessments were conducted independently by two authors. In cases of discrepancies, a third author was consulted. The data collected included study information, patients’ characteristics, diagnostic tools and criteria for frailty, number of frail patients at admission, follow-up durations, reported outcomes, and variables adjusted in the multivariate regression model for estimating the association between frailty and outcomes after KT. The Newcastle-Ottawa Scale (NOS) was employed to evaluate the quality of studies based on factors such as participant selection, group comparison, and validity [15]. The study quality system assigns a higher quality rating to studies with a greater number of stars.

### Statistical analyses

In this meta-analysis, the association between frailty and adverse outcomes following kidney transplantation was summarized using the risk ratio (RR) and corresponding 95% confidence interval (CI). For studies employing multivariate analysis, the RR and corresponding 95% CI were either directly extracted or calculated based on p-values. The variance was stabilized and normalized through logarithmic transformation. The study heterogeneity was assessed using Cochrane Q test and I^2^ statistics [16], with between-study heterogeneity classified as mild (I^2^ < 25%), moderate (I^2^ 25%~75%), and high (I^2^ > 75%) based on the Cochrane Handbook [14]. The random-effects model was augmented with heterogeneity to amalgamate the outcomes [14]. A sensitivity analysis was conducted to scrutinize the effect of each study on the overall results by excluding one study at a time [17]. Furthermore, subgroup analyses were carried out to evaluate the impact of study variables on the outcomes, including the assessment methods for frailty and follow-up durations, provided that adequate datasets (minimum of ten) were available. To visually assess the symmetry of the funnel plots, Egger’s regression asymmetry test was employed to estimate publication bias [18]. In our analyses, RevMan (version 5.1; Cochrane Collaboration, Oxford, UK) and Stata (version 12.0; Stata Corporation, College Station, TX) were used.

## Results

### Studies obtained

**Fig. 1:**
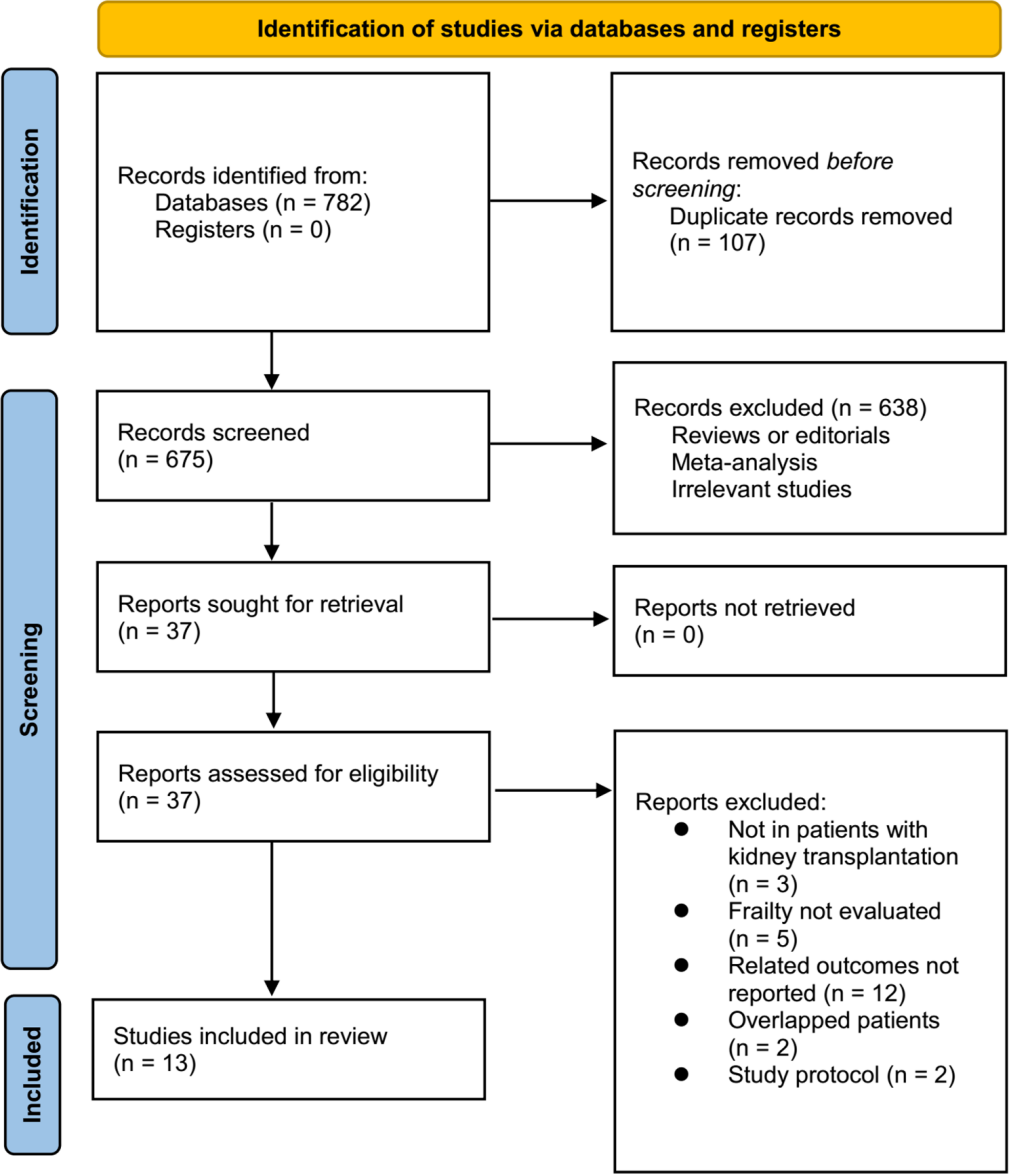
A summary of the literature search and study identification process;

As depicted in Fig. 1, the initial search yielded a total of 782 articles, of which 675 were retained after eliminating duplicate records. Furthermore, 638 articles were excluded from the meta-analysis due to their titles and abstracts being irrelevant to the subject matter, resulting in 37 studies being subjected to full-text analysis. Upon conducting a thorough review of the full-text, 24 studies were deemed unsuitable, leaving 13 eligible studies [19–31] for the meta-analysis. The rationale behind the exclusion of the 24 studies is presented in Fig. 1.

### Characteristics of the included studies

As shown in Tables 1 and 12 prospective [19–24, 26–31] and one retrospective [25] cohort studies were included in the meta-analysis. These studies were published between 2012 and 2022 in the United States, the Netherlands, Brazil, and Spain. All the studies included patients with ESRD who were admitted for KT. The number of patients of the included studies was 60 to 1113. The mean ages were 44 to 63 years, and the proportions of men were 53 to 71%. Physical Frailty Phenotype (PFP) was the mostly used assessment tool for frailty, which were used in eight studies [19–22, 24, 27, 30, 31]. Other evaluating tools for frailty were also used, such as short physical performance battery (SPPB) [23], Frailty Risk Score (FRS) [25], the Groningen Frailty Indicator (GFI) [26], the Inflammatory-Frailty Index (IFI) [29], and the new physical frailty phenotype (nPFP) [28]. Overall, 6279 patients from 13 cohort studies were included, and 1435 patients (22.9%) were with frailty before KT. The follow-up durations were from within hospitalization to 6.3 years after KT. As for the outcomes, mortality after KT was reported in eight studies [20, 22–24, 28–31], incidence of DGF in two studies [19, 31], postoperative complications in two studies [26, 27], and proportions of patients with longer hospitalization in three studies [21, 24, 25]. For all of the included studies, age was adjusted when the association between frailty and clinical outcomes after KT was estimated. In addition, for 12 of the included studies, other potential confounding factors, such as sex, ethnicity, etiology of ESRD, comorbidities, donor type, and surgical characteristics including cold ischemia time were also adjusted [19–24, 26–31]. In this review, the studies received a NOS of 7 to 9, indicating that they were of good quality overall (Table 2).

**Table 1:** Characteristics of the included studies

Study	Location	Design	Number of patients	Mean age (years)	Male (%)	Timing of frailty assessment	Tools for the diagnosis of frailty	Number of patients with frailty	Follow-up duration	Outcomes reported	Variables adjusted
Garonzik 2012	USA	PC	183	53	63.7	Immediately prior to surgery	PFP	46	Within hospitalization	DGF	Age, BMI, ethnicity, DM, donor type, CIT, and preemptive KT
McAdams 2015	USA	PC	537	53.2	60	At admission for KT	PFP	107	2.7 years	Mortality	Age, sex, ethnicity, DM, time on dialysis and preemptive KT, donor type, and CIT
McAdams 2017	USA	PC	589	NR	NR	At admission for KT	PFP	141	Within hospitalization	LOS	Age, sex, ethnicity, DM, BMI, year on dialysis, donor type and characteristics, and CIT
Nastasi 2018	USA	PC	719	51.6	62.3	At admission for KT	SPPB	336	5 years	Mortality	Age, sex, ethnicity, BMI, years on dialysis, cause of ESRD, donor type, CVD, lung disease, and DM
Konel 2018	USA	PC	773	54	62.2	At admission for KT	PFP	126	5 years	Mortality	Age, sex, ethnicity, education, BMI, cause of ESRD, time on dialysis, modified CCI, and donor type.
Schaenman 2019	USA	RC	60	52.2	65.2	At admission for KT	FRS	17	Within hospitalization	LOS	Age
Schopmeyer 2019	The Netherlands	PC	150	51.8	62.6	At admission for KT	GFI	23	30 days	POC	Age, CCI, smoking, duration of dialysis, type of transplantation, and re-transplantation
Chu 2019	USA	PC	569	51.7	60.8	At admission for KT	PFP	91	4 years	LOS and mortality	Age, sex, ethnicity, BMI, DM, year on dialysis, cause of ESRD, donor type, and DGF
Mantovani 2020	Brazil	PC	87	44.8	53.1	At admission for KT	PFP	32	Within hospitalization	POC	Age, sex, ethnicity, cause of ESRD, BMI, CVD, DM, year of dialysis, and donor type
Haugen 2021	USA	PC	378	53	65.9	At admission for KT	IFI	152	5 years	Mortality	Age, sex, ethnicity, donor type, CCI, cause of ESRD, and smoking status
Chen 2021	USA	PC	1113	52.9	60.4	At admission for KT	nPFP	190	6.3	Mortality	Age, sex, ethnicity, donor type and CCI
Jose 2022	Spain	RC	296	62.6	70.6	At admission for KT	PFP	79	1.9	Mortality	Age, sex, HTN, DM, CAD, HF, PAD, year of dialysis, SCr at 1 year
Parajuli 2022	USA	PC	825	55.3	60	At admission for KT	PFP	95	2.8	DGF and mortality	Age, sex, ethnicity, cause of ESRD (DM vs. other), previous transplant, living donor, depleting induction, pre-transplant dialysis duration

PC, prospective cohort; RC, retrospective cohort; KT, kidney transplantation; DGF, delayed graft function; LOS, length of hospital stay; POC, postoperative complications; NR, not reported; BMI, body mass index; DM, diabetes mellitus; ESRD, end-stage renal disease; CVD, cardiovascular disease; HTN, hypertension; CAD, coronary artery disease; HF, heart failure; PAD, peripheral artery disease; SCr, serum creatinine; CIT, cold ischemia time; SPPB, short physical performance battery; TUGT, Timed Up and Go Test; CCI, Charlson comorbidity index; FRS, Frailty Risk Score; GFI, Groningen; Frailty Indicator; IFI, Inflammatory-Frailty Index; nPFP, new physical frailty phenotype; PFP, Physical Frailty Phenotype;

**Table 2:** Study quality evaluation via the Newcastle-Ottawa Scale

Study	Representativeness of the exposed cohort	Selection of the non-exposed cohort	Ascertainment of exposure	Outcome not present at baseline	Control for age	Control for other confounding factors	Assessment of outcome	Enough long follow-up duration	Adequacy of follow-up of cohorts	Total
Garonzik 2012	1	1	1	1	1	1	1	1	1	9
McAdams 2015	1	1	1	1	1	1	1	1	1	9
McAdams 2017	1	1	1	1	1	1	1	1	1	9
Nastasi 2018	1	1	1	1	1	1	1	1	1	9
Konel 2018	1	1	1	1	1	1	1	1	1	9
Schaenman 2019	0	1	1	1	1	0	1	1	1	7
Schopmeyer 2019	1	1	1	1	1	1	1	1	1	9
Chu 2019	1	1	1	1	1	1	1	1	1	9
Mantovani 2020	1	1	1	1	1	1	1	1	1	9
Haugen 2021	1	1	1	1	1	1	1	1	1	9
Chen 2021	1	1	1	1	1	1	1	1	1	9
Jose 2022	0	1	1	1	1	1	1	1	1	8
Parajuli 2022	1	1	1	1	1	1	1	1	1	9

### Primary outcome

**Fig. 2:**
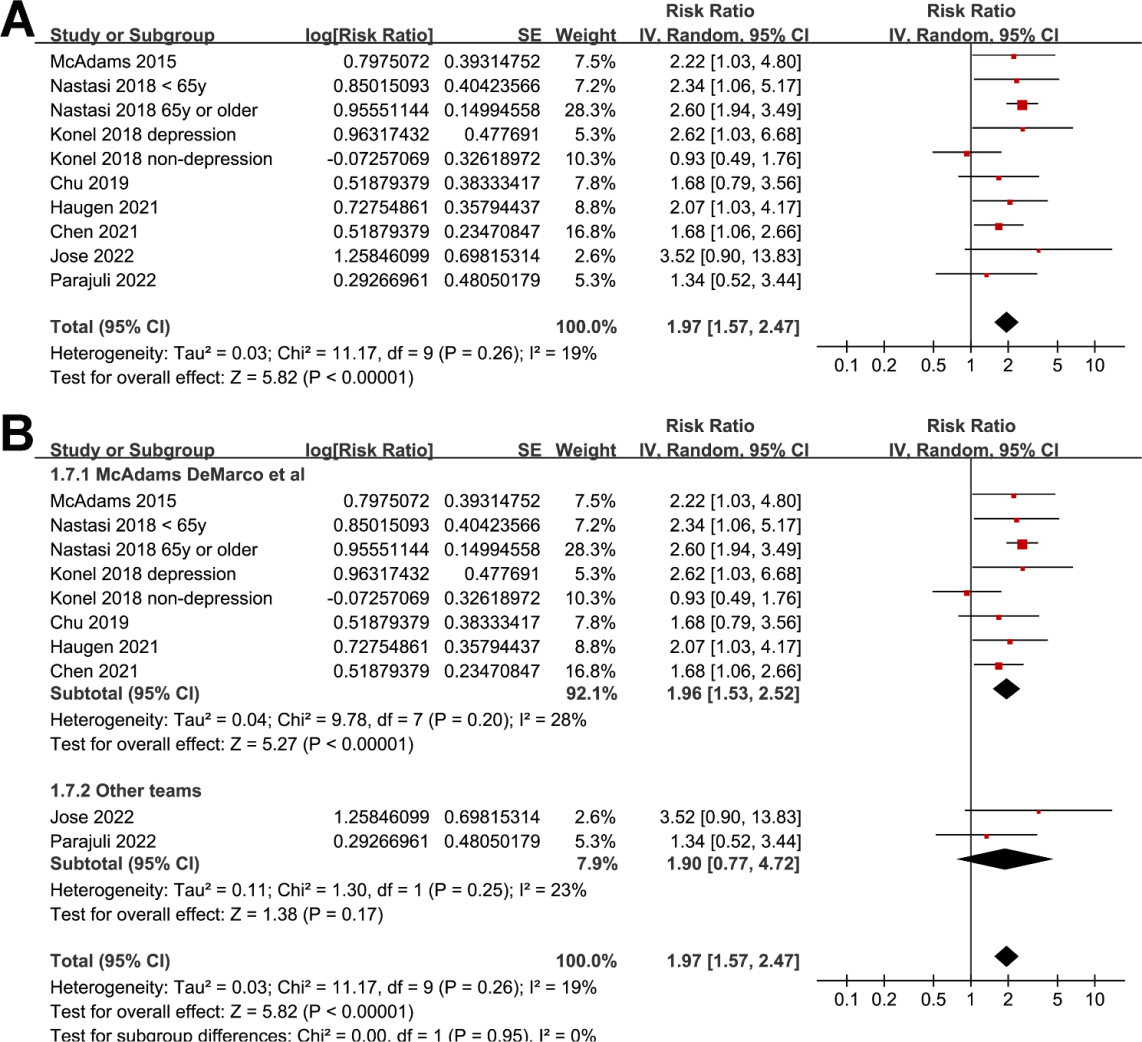
Forest plots for the meta-analyses regarding the association between frailty and all-cause mortality after KT. **A**, forest plots for the overall meta-analysis; and **B**, subgroup analyses according to the research teams;

**Fig. 3:**
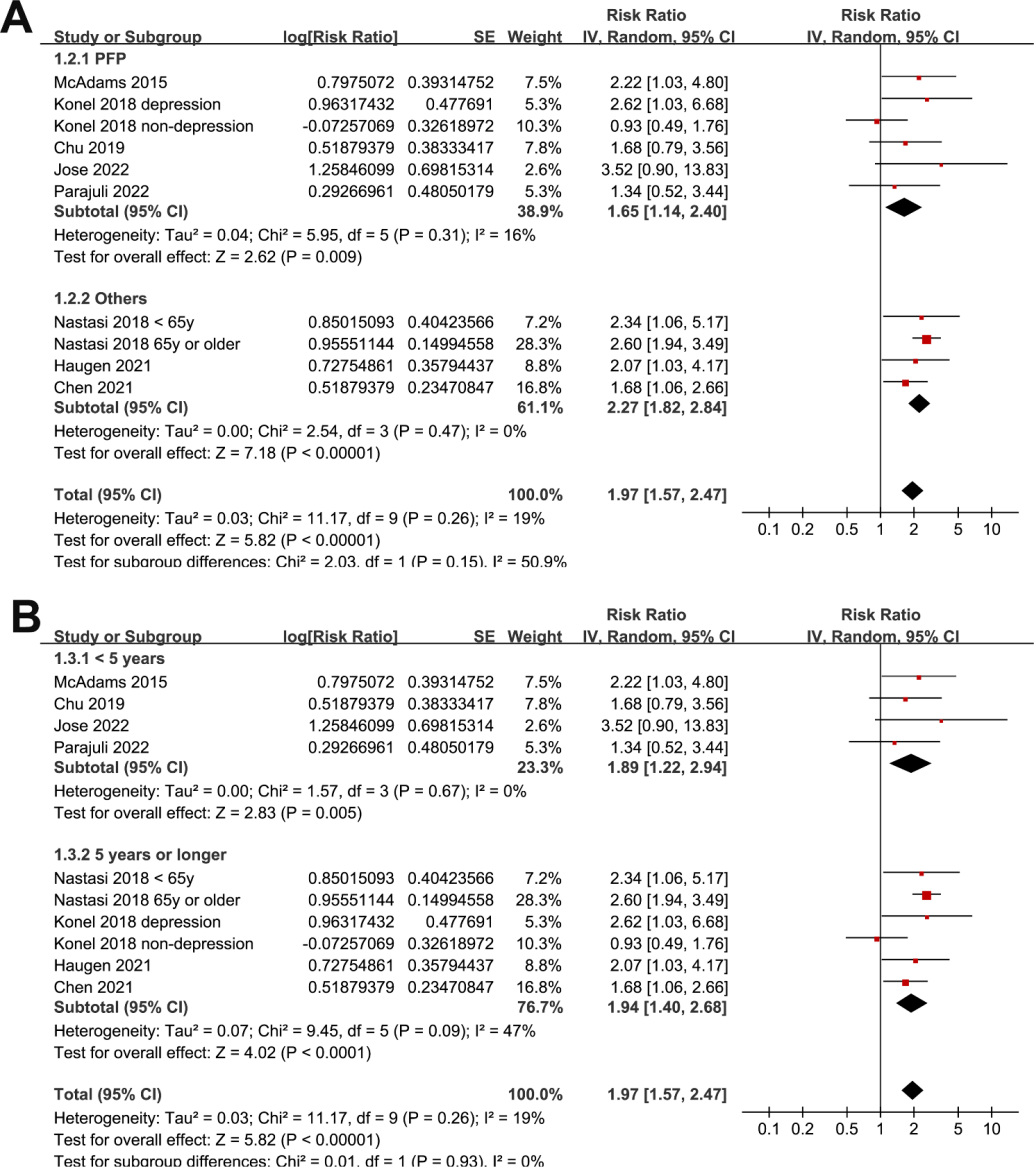
Forest plots for the subgroup analyses regarding the association between frailty and all-cause mortality after KT. **A**, subgroup analyses according to the evaluating tool of frailty; and **B**, subgroup analyses according to the follow-up duration;

Eight studies [20, 22–24, 28–31] reported the association between frailty and all-cause mortality after KT. Since one study reported the outcomes according to age [23], and another study reported the outcomes according to the depressions status of the patients [22], these datasets were included into the meta-analysis independently. Overall, ten datasets from [20, 22–24, 28–31] were available for the meta-analysis of the association between frailty and all-cause mortality after KT. Mild heterogeneity was observed (Cochrane Q test = 0.26, I^2^ = 19%). Pooled results showed that compared to those without frailty, frail patients at admission had a higher incidence of mortality (risk ratio [RR]: 1.97, 95% confidence interval [CI]: 1.57 to 2.47, p < 0.001; Fig. 2A). Sensitivity analyses by excluding one study at a time showed consistent results (RR: 1.74 to 1.21, p all < 0.05). Since we noticed that substantial of the included studies were performed by the team of McAdams DeMarco et al. To account for this unequal distribution, a subgroup analysis was performed accordingly, which showed similar results in studies by the team of McAdams DeMarco et al. and in studies of other groups (p for subgroup = 0.95; Fig. 2B). In addition, subgroup analysis suggested the association between frailty and high mortality risk after KT was consistent in studies of frailty assessed via PFP or other methods (p for subgroup = 0.15; Fig. 3A), and in studies of follow-up duration < or ≥ 5 years (p for subgroup = 0.93; Fig. 3B).

### Secondary outcomes

**Fig. 4:**
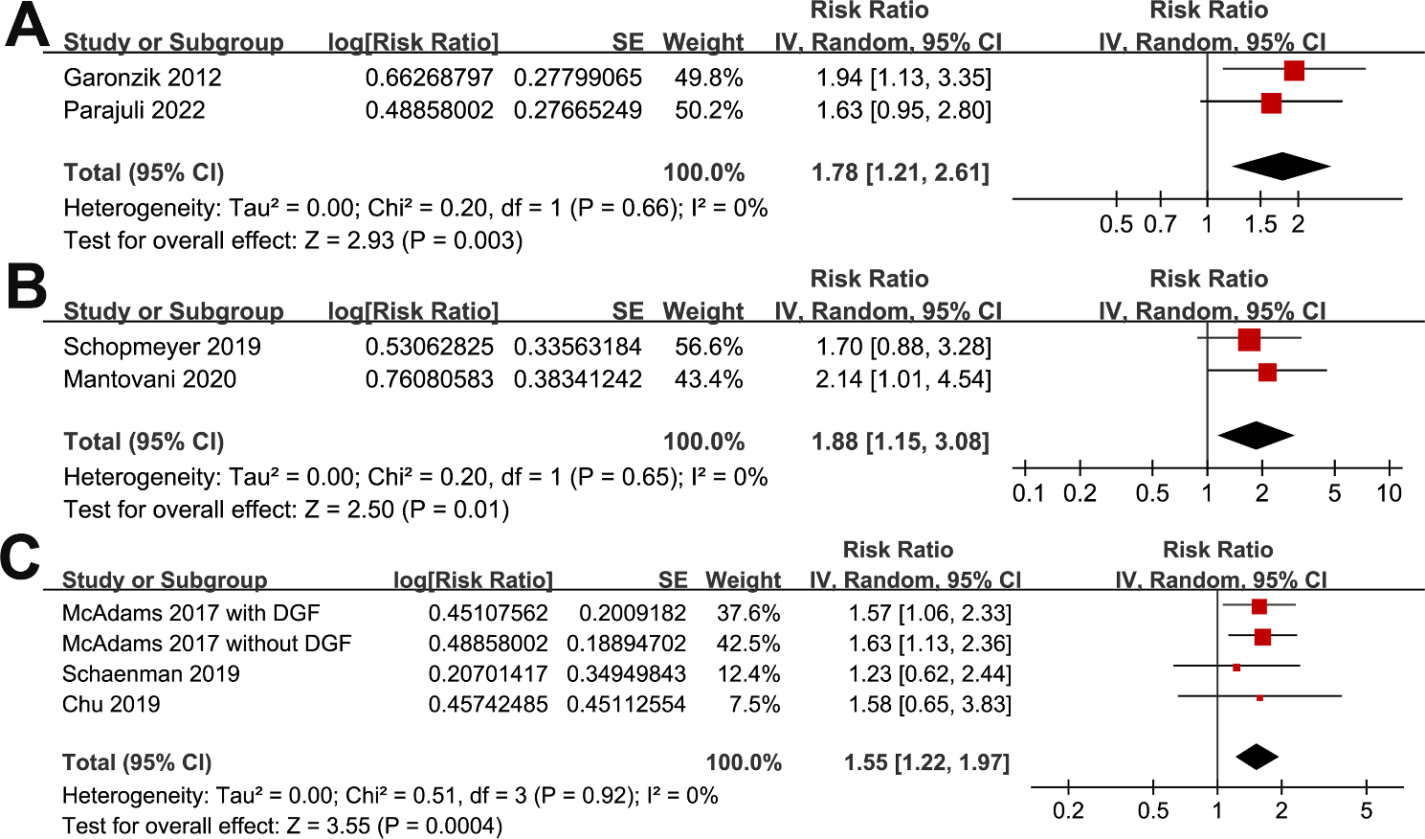
Forest plots for the meta-analyses of the secondary outcomes. **A**, forest plots for the association between frailty and incidence of DGF after KT; **B**, forest plots for the association between frailty and incidence of postoperative complications after KT; and **C**, forest plots for the association between frailty and longer hospitalization after KT;

In addition, meta-analyses with two to four datasets showed that compared to those without frailty, frail patients at admission had higher incidence of DGF (RR: 1.78, 95% CI: 1.21 to 2.61, p = 0.003; I^2^ = 0%; Fig. 4A), postoperative complications (RR: 1.88, 95% CI: 1.15 to 3.08, p = 0.01; I^2^ = 0%; Fig. 4B), and longer hospitalization (RR: 1.55, 95% CI: 1.22 to 1.97, p < 0.001; I^2^ = 0% Fig. 4C).

### Publication bias

**Fig. 5:**
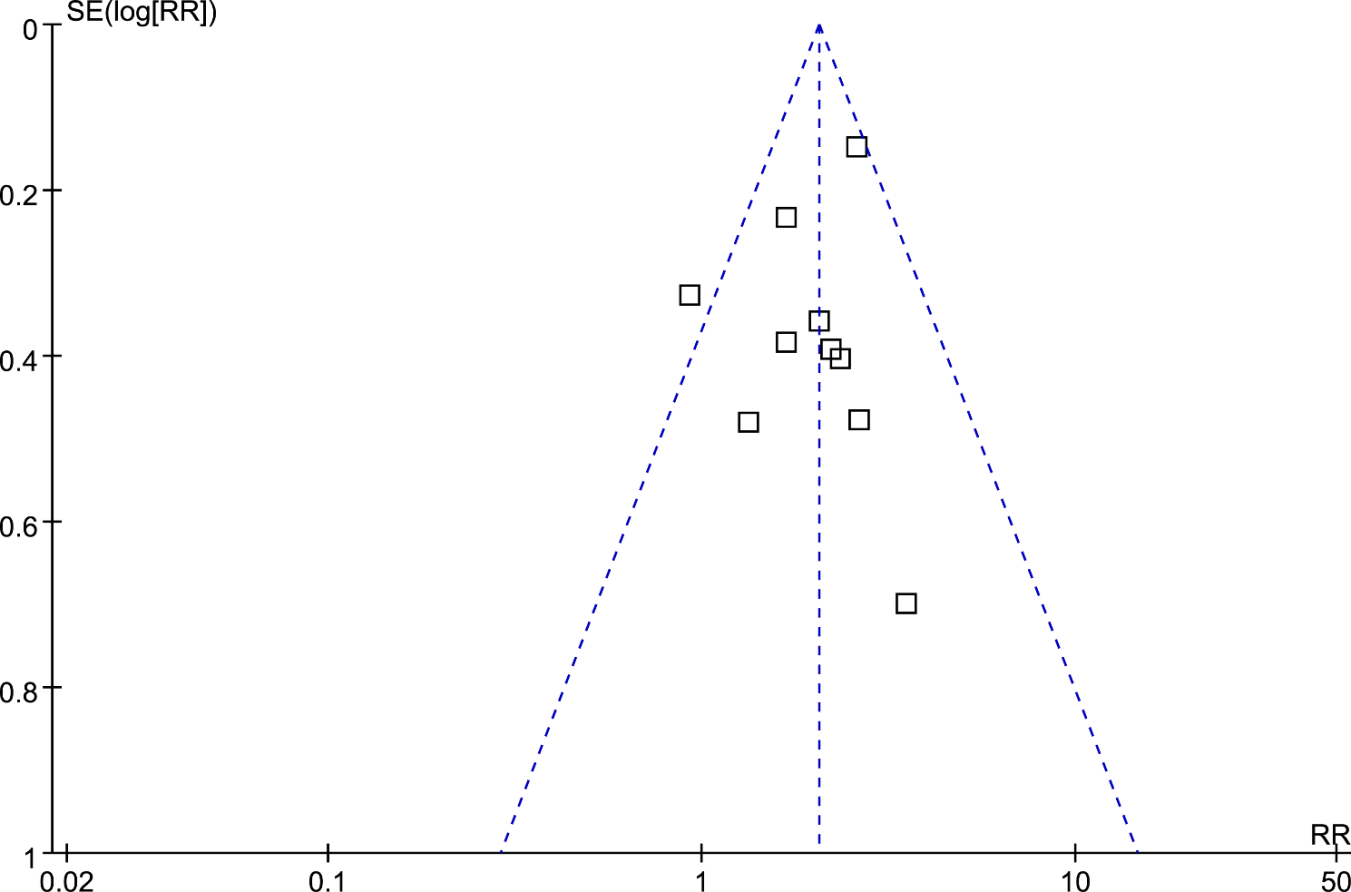
Funnel plots for the publication bias underlying the meta-analysis of the association between frailty and all-cause mortality after KT;

Based on the findings presented in Fig. 5, the funnel plots for the meta-analysis examining the correlation between frailty and all-cause mortality in patients following KT exhibit symmetry, suggesting that the risk of publication bias is low. Furthermore, the results of Egger’s regression test indicate a low risk of publication bias (p = 0.61). However, due to the limited number of studies included, it was not possible to determine the publication biases underlying the meta-analyses for the other outcomes.

## Discussion

This systematic review and meta-analysis synthesized the outcomes of 13 eligible cohort studies, revealing a significant correlation between frailty at admission and heightened mortality risk following KT. Furthermore, frailty in KT patients may also increase the likelihood of DGF and postoperative complications, as well as prolong hospitalization. Collectively, these results suggest that frailty may serve as a crucial prognostic indicator for KT patients.

To the best of our knowledge, only one meta-analysis has been published previously to evaluate the prevalence and related factors of frailty in patients with KT [10]. This meta-analysis included 14 observational studies and showed that the pooled overall prevalence of frailty was 17.1% among kidney transplant candidates before transplantation [10]. It was also shown that frailty before transplantation was correlated with higher age, lower rate of preemptive transplantation, longer duration of DGF, and length of stay longer than 2 weeks [10]. Of note, the primary outcome of the current meta-analysis is different from that of the previous meta-analysis by Quint et al. [10]. The primary outcome of our meta-analysis was to determine the prognostic role of frailty on prognosis of recipients of KT. Accordingly, studies including candidates of KT who were not eventually received the transplant were excluded in our meta-analysis. More importantly, we found that frailty at admission was a predictor of mortality in patients after KT. The new information from our meta-analysis is that frailty is not only prevalent in candidates of KT, but also a prognostic factor for the mortality risk of patients after KT. The present meta-analysis exhibits several methodological strengths that warrant attention. Firstly, the inclusion of solely cohort studies facilitates the establishment of a longitudinal association between frailty and unfavorable prognosis of patients following KT. Furthermore, the pooling of multivariate-adjusted data from all the studies included for the mortality outcome suggests that the correlation between frailty and heightened mortality risk after KT may be autonomous of patient attributes such as age, sex, etiology of ESRD, and comorbidities, among others. Ultimately, in meta-analyses with sufficient datasets, we conducted numerous sensitivity and subgroup analyses to confirm the robustness of the results. Collectively, the outcomes of the meta-analysis indicate that frailty upon admission may serve as an autonomous prognostic factor for mortality risks following KT in admitted patients. These findings lend support to the use of frailty assessment as a prognostic tool for patients undergoing KT.

The mechanisms underlying the association between frailty and high mortality risk in patients after KT may be multifactorial. As indicated in the secondary outcomes of the meta-analysis, frailty has been associated with higher incidence of DGF, which may be related to a long-term risk of graft failure [32]. In addition, results of the meta-analysis also showed that frailty is associated with increased incidence of postoperative complications after KT, and some of the severe complications may also adversely affect the long-term efficacy of the graft and survival of the patients, such as vascular complications [33]. Moreover, there are several studies which suggested that frailty was associated with poor adherence to immunosuppressive therapy after KT [34, 35], which may also lead to graft failure and increased mortality risk in this population. Studies are warranted to determine other potential mechanisms underlying the association. Although we found that frailty may be a risk factor of poor prognosis of patients after KT, these findings do not mean that KT should not be performed in frail patients. In fact, several recent studies indicated that the frailty status of the patients with ESRD could be improved after KT [36–39]. On the hand, efforts should be made to determine if multimodal prehabilitation in frail patients before KT could improve the long-term survival and other clinical outcomes of the patients [40].

This meta-analysis is not without limitations. Specifically, the heterogeneity of evaluating tools for frailty among the included studies may have impacted the results. However, it is important to note that there is currently no consensus on the gold-standard method for defining frailty in clinical practice or in patients with KT. As such, future studies are necessary to establish the optimal tools and cutoffs for identifying frailty in these populations. Among these scales, the Clinical Frailty Scale (CFS) is also clinically practical [41, 42]. It categories into different levels of frailty based on their functional status and level of independence [41, 42]. The CFS ranges from 1 (very fit) to 9 (terminally ill), with various levels in between. It takes into account factors such as mobility, self-care abilities, and cognitive function [41, 42]. This scale can also be helpful in assessing the overall frailty of patients considering KT and studies are warranted in the future to determine the prognostic efficacy of CFS defined frailty in patients after KT. Subsequently, a paucity of studies was accessible for the secondary outcomes, and it is imperative that the outcomes are authenticated in extensive prospective cohort studies. Furthermore, despite the utilization of multivariate analyses in all the incorporated studies to evaluate the correlation between frailty and the prognosis of patients after KT, the likelihood of residual factors that may confound the association cannot be entirely ruled out. Furthermore, due to the nature of this meta-analysis of observational studies, it was not possible to establish a definitive causal relationship between frailty and adverse prognostic outcomes for patients following KT. Therefore, it is recommended that clinical studies be conducted to investigate the potential impact of enhancing frailty status prior to surgery on the clinical outcomes of KT. Finally, the situation of individual candidate of KT is different and unique, and the decision to proceed with a KT should be made on a case-by-case basis. Frailty scores are just one of many factors that medical teams consider when evaluating a patient’s candidacy for transplantation.

## Conclusion

In conclusion, results of the meta-analysis indicate that frailty may be a risk factor of all-cause mortality, delayed graft function, postoperative complications, and longer hospitalization in patients after KT. Studies are warranted to determine the optimal evaluating tool for the diagnosis of frailty in these patients, and to explore whether improving the frail status of the patients before surgery could improve the prognosis of patients after KT.

## Data Availability

All data generated and analyzed during this study are included within the published article.
